# Investigating interactive methods in remote chestfeeding support for lactation consulting professionals in Brazil

**DOI:** 10.3389/fdgth.2023.1143528

**Published:** 2023-04-03

**Authors:** Jessica de Souza, Cinthia Calsinski, Kristina Chamberlain, Franceli Cibrian, Edward Jay Wang

**Affiliations:** ^1^ Department of Electrical and Computer Engineering, University of California San Diego, San Diego, CA, United States; ^2^ Independent Researcher, Sao Paulo, Brazil; ^3^ Division of Extended Studies, University of California San Diego, San Diego, CA, United States; ^4^ Fowler School of Engineering, Chapman University, Orange, CA, United States

**Keywords:** remote care, chestfeeding, lactation consultant, human–computer interaction, chestfeeding support

## Abstract

**Objective:**

Lactation consultants (LCs) positively impact chestfeeding rates by providing in-person support to struggling parents. In Brazil, LCs are a scarce resource and in high demand, risking chestfeeding rates across many communities nationwide. The transition to remote consultations during the COVID-19 pandemic made LCs face several challenges to solve chestfeeding problems due to limited technical resources for management, communication, and diagnosis. This study investigates the main technological issues LCs have in remote consultations and what technology features are helpful for chestfeeding problem-solving in remote settings.

**Methods:**

This paper implements qualitative investigation through a contextual study (n=10) and a participatory session (n=5) to determine stakeholders’ preferences for technology features in solving chestfeeding problems.

**Findings:**

The contextual study with LCs in Brazil characterized (1) the current appropriation of technologies that help during consultations, (2) technology limitations that affect LCs’ decision-making, (3) challenges and benefits of remote consultations, and (4) cases that are easy and difficult to solve remotely. The participatory session brings LCs’ perceptions on (1) components for an effective remote evaluation, (2) preferred elements by professionals when providing remote feedback to parents, and (3) feelings about using technology resources for remote consultations.

**Conclusion:**

Findings suggest that LCs adapted their methodologies for remote consultations, and the perceived benefits of this modality show interest in continuing to provide remote care as long as more integrative and nurturing applications are offered to their clients. We learned that fully remote lactation care might not be the main objective for overall populations in Brazil, but as a hybrid mode of care that benefits parents by having both modalities of consultations available to them. Finally, remote support helps reduce financial, geographic, and cultural barriers in lactation care. However, future research must identify how generalized solutions for remote lactation care can be, especially for different cultures and regions.

## Introduction

There is extensive literature demonstrating the benefits of exclusive chestfeeding for both mother and child (1–5). Additionally, chest milk is a free, natural renewable source of food that requires no packaging or storage (6). In Low and Middle-Income Countries (LMICs), encouragement of chestfeeding is even more needed as it can be critical for a baby’s survival, especially in low-income families that have limited access to formula and rely on human milk as a free source of nutrition for neonates (7).

Despite compelling evidence on the benefits of exclusive chestfeeding, only 44% of infants globally meet the WHO’s recommendation of exclusive chestfeeding until the age of 6 months (8). In Brazil, the prevalence of exclusive chestfeeding among infants between 4 months and 6 months is 59% and 45%, respectively (9). Some of the reasons that explain this low adherence include insufficient milk supply, chestfeeding-related fatigue, medical conditions in the baby or mother, difficulties with feeding techniques or pain (10), lack of support from family (11), and lack of access to expert lactation support (12). Additional reasons for premature chestfeeding cessationin underdeveloped regions include lack of access to chestfeeding information, gendered childcare workloads, and body shaming due to the exposure of breasts (7).

Another impacting factor on chestfeeding numbers is the type of birth delivery. Cesarean birth (C-section) impacts the initiation and duration of chestfeeding compared to natural vaginal birth (13). Brazil is one of the leading countries in cesarean births, a delivery modality often scheduled in advanced private clinics or a procedure that is sometimes forced on parents in the public health system (14, 15). During prenatal consultations, parents must be educated about pregnancy care, delivery options, reproductive health, and chestfeeding before the baby’s arrival. Still, a study showed that 40% of women did not receive instructions about how to chestfeed at any moment pre- or post-birth (16). The need for professional training often causes this gap in providing information to parents in the Brazilian healthcare system and missing hospital policies that regulate clinical practices in chestfeeding according to the World Health Organization guidelines (17).

A lactation consultant (LC) is a professional who promotes chestfeeding education and provides support to parents. LCs are specialized in chestfeeding, milk supply, breast and nipple issues, baby sleep, preparing the mothers for milk management before their return to work, and prenatal education (18). LCs play a key role in helping mothers transition into chestfeeding, making it easier and painless and increasing the possibility of continued chestfeeding through 6 months or longer (19, 20). Unfortunately, there is limited availability of International Board Certified Lactation Consultants (IBCLCs) around the globe. For example, in 2021, there were in total 3.6 million births in the US and only 18.5k LCs with IBCLC certification, a rate of 194 babies per LC a year (21, 22). Meanwhile, in Brazil, in the same year, there were a total of 2.6 million births for a total of 154 certified LCs, a rate of 16,883 babies per LC a year (21, 23). The high demand for LC professionals is notable, especially in LMICs such as Brazil, where parents may have more difficulties finding LCs for chestfeeding guidance. Therefore, it is crucial to develop better tools for LC that allow them to provide more immersive and supportive experiences for the parents (24–26).

Traditionally, LC work is mostly performed in-person since it requires closer observation and even physical touch for the mother and baby assessment. In traditional in-person lactating care, LCs are commonly present with their patients from the first minute of the baby’s life, providing initial chestfeeding guidance to the mother and often through a couple of days or weeks depending on the clinical case (27). Their in-person work is very immersive and can be hands-on, with LCs sometimes helping the mother to attach the baby to the breast properly or showing the mother how to reposition the baby to avoid choking. In addition, LCs perform a physical evaluation of the mother’s breasts, examine the baby’s internal mouth structure, assess breast engorgement, introduce laser therapy for nipple healing (10), and use dolls and breast plushies with internal anatomy (28) to educate the mother in how milk production happens and how positioning affects milk extraction.

With the high adoption of smartphones by the Brazilian population and WhatsApp being a leader communication app in the nation (29), some public health facilities in urban areas such as family clinics started in 2018 to provide patient services such as appointment scheduling, health orientations, and vaccine campaign notifications (30). The app WhatsApp became a popular communication tool between patients and healthcare providers, including chestfeeding education and family support with neonates (31, 32).

When LCs transitioned to virtual consultations due to the COVID-19 pandemic, they incorporated into their workplace apps to which the population is already familiar with, such as WhatsApp, Instagram, and Facebook. LCs needed to remodel their approach to accommodate the mother’s needs with the same quality but without the resources from in-person consultations (18, 33). Lactation care and other modalities that require physical evaluation, like physical therapy, require reinvention to achieve patient satisfaction and solve cases when the scenario switched from in-person to remote (18). As such, LCs used communication and social media apps to provide remote consultations, and educate parents using features these apps provide and having larger visibility (18, 34).

On the one hand, remote lactation care has faced challenges such as a limited field of view of the patient (mother and baby) during video calls, issues with having instructions understood by the patient, mothers with difficulty in demonstrating their chestfeeding problems, lack of standards and guidelines, and technical difficulties (26, 35, 36). On the other hand, remote lactation care brings benefits and advantages, such as helping combat the feeling of isolation, allowing the LC to provide just-in-time nurturing and reassurance to the mother during virtual consultations (25, 26), and enabling the LC to focus on effective communication, which benefits the patient’s independent learning (35) since it requires higher engagement between patient and LC during consultations. This independent learning positively impacts mothers’ intentions in exclusive chestfeeding up to 6 months and reduces the risk of chestfeeding cessation at 3 months by 25% (37).

Particularly in the Global South, remote healthcare takes advantage of the accessibility of mobile technologies that can play a key role in broadening access to healthcare and connecting patients and doctors even in remote areas, adding tools for community health workers, improving professional decision-making, and providing more resources for patients (38–42). However, in Latin America, the adoption of telemedicine is being impacted by physician resistance in both public and private sectors, lack of technological infrastructure, professional training, and financial support by government entities (43). Additionally, it is an established practice in the field that the use of technologies during chestfeeding should be limited and not necessarily relied upon to promote strong human bonds between the parent and child, such as eye contact and touch (44). For these reasons, it is necessary to understand the opportunities and barriers faced by lactation consultants using telemedicine in the LMIC context in order to develop appropriate technologies that make more accessible remote consultations without invalidating the human experience for the mother and at the same time improving the overall efficacy of the care provided.

Our research seeks to understand the work dynamics of LCs during remote consultations, their interactions with technology as healthcare professionals, the challenges and opportunities that remote consultations offer in their practice, their general routine as LCs, and how they provide parental support. The main questions asked in this research lie in (1) What is the process of LCs in identifying chestfeeding problems remotely, (2) what are the technical elements of communication they use for problem-solving, and (3) how do they exploit technology for a more immersive, didactic, and empathetic communication with their patients. We hypothesize that some technical elements used remotely are more successful in solving specific chestfeeding problems than others. The study contains two parts for this investigation: (1) a contextual study consisting of in-depth interviews with LCs for a detailed understanding of their work and (2) a participatory workshop consisting of a focus group interview with LCs using video provocations to stimulate group discussion and technology probes to identify elements that make problem-solving easier.

## Methods

To better understand the routine, technical approach, and needs of LCs during remote consultations, we designed a qualitative study that contains two parts. First, we conducted a contextual study consisting of in-depth semi-structured interviews with each LC. Second, we conducted participatory workshops consisting of focus groups, video provocations, and technology probes for chestfeeding problem-solving.

### Contextual study

We conducted a contextual study to identify the work approach of LCs, the challenges and benefits of remote consultations in their careers, and the role of technology in lactating care. We conducted semi-structured interviews split into two major themes: context and remote work. The interviews were prompted by 12 main questions (see details in Table 1) that led to deeper discussions. Questions were previously shared with participants through WhatsApp for reflection before interviewing via video calls. During the call, the participants were made aware of the project’s goals and asked permission to take notes of their responses. The interviews lasted an average of 45 minutes per person, totaling close to 6 hours of information plus the three interviews through text and audio recordings.

**Table 1:** Prompt questions used for the contextual study.

Category	Questions
Context	What is your current role and what are your specialties within this role?
	How long have you worked as a lactation consultant?
	Are you IBCLC certified? Since when?
	What is your current work setting?
Remote work	Did you start having remote consultations before or because of the COVID-19 pandemic?
	Do you like providing remote consultations? What benefits did you notice from it?
	What are the biggest challenges you have when providing remote care?
	What apps/software do you use to contact your clients for remote consultations?
	What are the most common questions you receive from patients through remote settings?
	What are the easiest cases to solve remotely? What about the hardest cases?
	How many remote consultations per week do you have on average?
	If you could change anything to improve remote lactating care, what would it be?

### Participatory workshops

**Figure 1:**
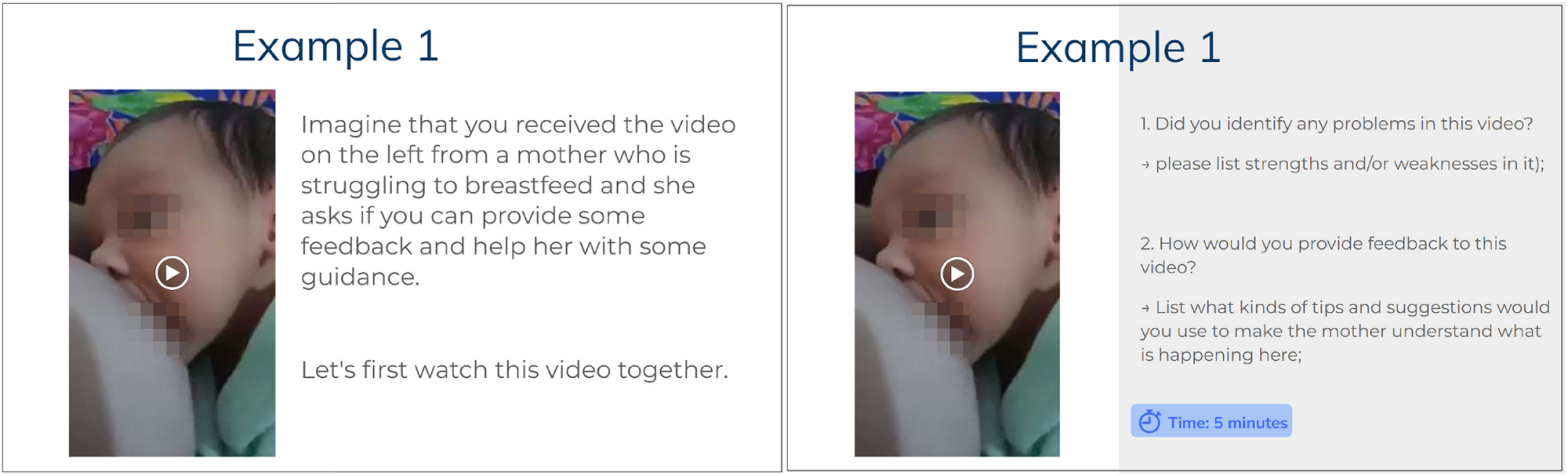
Video provocation slides used in the focus group. Participants were asked to imagine themselves receiving the following video from their patients (left). Then, some included questions started a discussion on how LCs would provide feedback and guidance for the mother if problems were found (right).

**Figure 2:**
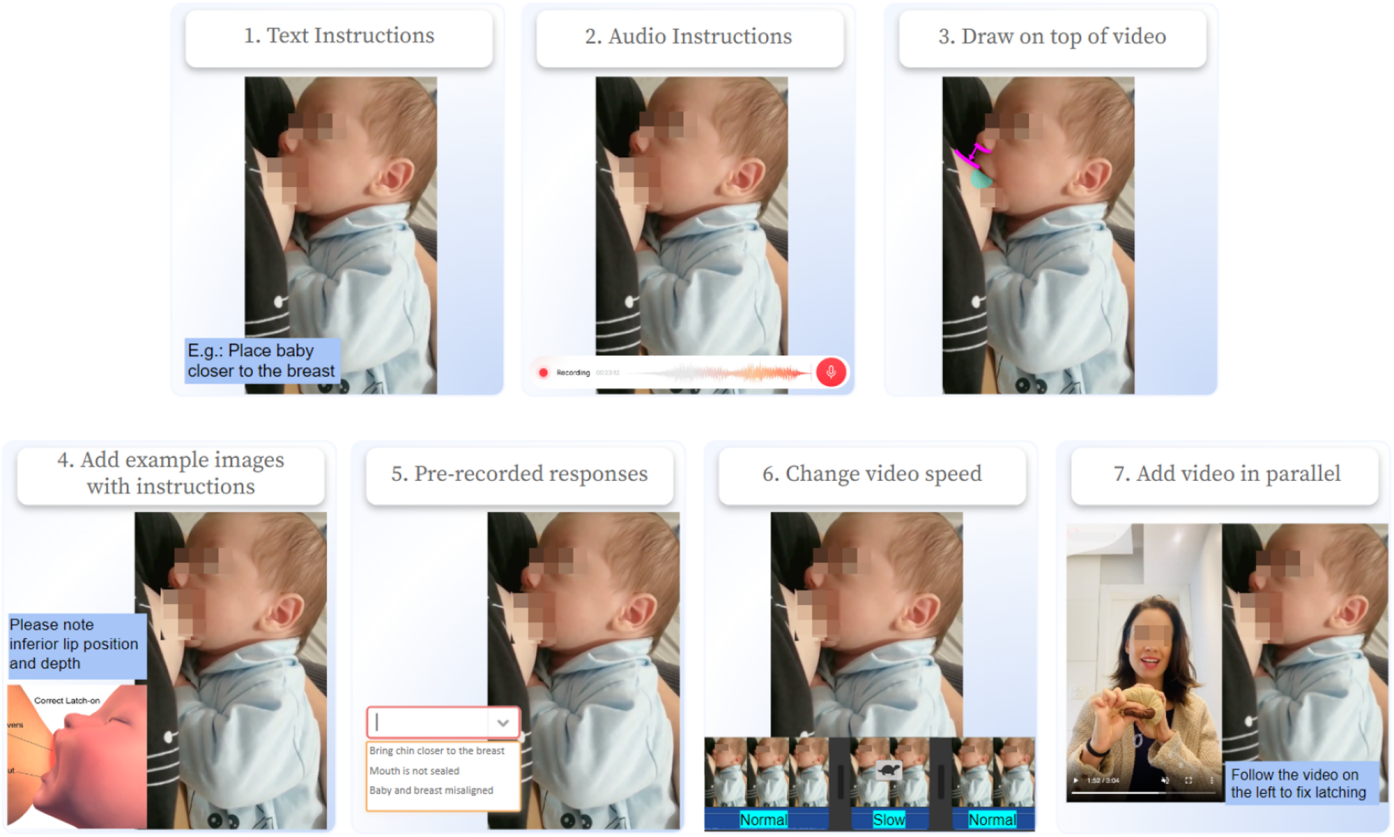
Features suggested in technology provocation. A total of seven options were given to participants in the provocation: (1) text instructions, (2) audio instructions, (3) drawing on the top of video, (4) adding in parallel to video example images, (5) using pre-recorded responses for text, (6) changing video speed, or (7) adding video in parallel to the original one.

To supplement our understanding of LC remote work and encourage and motivate the LC to envision software applications to support their work, we conduct the following activities:

• **Focus group**: To better understand LC remote work, we conducted a focus group. We started by explaining the study goals and getting the participants’ consent to have the focus group recorded before beginning the questions. The participants then introduced themselves and provided details of their specialty as LCs. During the focus group, we presented slides with two main activities, video provocation, and technology proof.
• **Video provocation**: The HCI community uses several tools and processes to understand the needs of underserved communities and the complexity of their everyday life activities (40). In this work, we used three real videos with chestfeeding cases the consultants face in routine consultations as an exploration artifact (45). The first video introduced a baby’s chestfeeding with ineffective milk extraction. It illustrated features like a limited field of view of the baby (to expose a technology limitation) and a shallow latch in the breast. The second video had a similar example to the first video with non-nutritive milk extraction. However, it showed a broader field of view, giving the participants more information about mother and baby positioning. The third video showed a baby with effective milk extraction but with possible changes in the baby’s latching and positioning and space for comments related to milk supply.
  For each video shown to the participants, the same text and questions were presented accordingly (see Figure 1 as an example), and we asked the LCs questions to encourage them to analyze the videos and provide comments with the richness of information and engagement to the group, as follows: “Did you identify any problems in this video?,” “Can you tell me the strengths or weaknesses of this video?,” “How would you provide feedback on this video in case you received it from one of your clients?,” “What elements in this list would you find useful to provide feedback for a better chestfeeding experience?,” “How would you use the element and why?.”
  Overall, these videos served as a starting point to bring the LCs to an environment that enabled their professional judgments and problem-solving skills in order to work together toward a solution to the problems found in these videos.
• **Technology probe**: We presented the participants with feature elements as technology probes (46), which served as a base for the discussion of what kinds of features in remote applications are useful and effective for lactation consultants to use in their remote care routines. We also used the videos to discuss how to solve such cases remotely. After that, lactation consultants had to imagine themselves with a tool with several features that they could use to create a new artifact using the original videos. This artifact would help provide feedback to mothers who needed guidance in proper chestfeeding. Finally, the group discussed what features they would use to solve the problem in the video, where each participant shared their feature preferences, how they would use it, and what information they would add to each feature.
  The feature elements presented to the lactation consultants in this part of the study are presented in Figure 2. The first element is (1) the text instruction element, which could be used for comments or step-by-step instructions; the second element is (2) the audio instruction feature, which enables voice over the original video, which could also be used for giving instructions, narrating what is happening, and informing potential issues. Some visual elements were also provided, such as (3) drawing on top of the original video to help give instructions and fix positioning, (4) adding example images parallel to the original video, or even (7) adding example videos with the correct example or tutorial to answer to questions. In addition, the option to use (5) pre-recorded responses was seen as optional for texts with instructions that are common to share among patients with more general cases, and (6) changing the video speed could be another option for emphasizing some video parts that need more attention than others. We also gave participants the option to suggest or add their own technological elements if interested and how they would use them in the same context as the others. Lastly, LCs were oriented toward the possibility of combining multiple elements to give feedback in a remote or semi-synchronous consultation.

### Participants

The participants for the contextual study were LCs who lived and worked in Brazil (n=10), in the states of Rio Grande do Sul, Sao Paulo, Santa Catarina, and Rio de Janeiro. All LCs perform remote consultations (RCs) with patients from their current states and also from other states. All the participants work as lactation consultants and are also specialized in other areas, such as obstetric nurses (n=7), physical therapists that prepare mothers for labor (n=2), and neonatal speech pathologists (n=1). The participant’s age ranged from 25 to 43 years (μ=36.90, σ=5.50). The average years of experience as lactation consultants ranged from 3 to 20 years (μ=8.50, σ=5.15). The participants work in-person in public and private hospitals, clinics, or their own offices as private consultants but also perform consultations remotely in their private modalities. Most of the LCs are also mothers (n=7), in which some of them became mothers before and during their studies to become an LC. Therefore, it should be noted that their perspectives come from an LC and mother standpoint. All participants have experience working remotely and are familiar with using smartphones and computers. Table 2 provides detailed information about the participants’ demographics.

**Table 2:** Participant’s demographics.

Category	Subcategory	Participants (N)	Mean	SD
Gender	Female	10		
Race/ethnicity	Hispanic/latino	10		
Age range (years)	25, 43		36.90	5.50
Age group	Less than 30	1		
	Between 30 and 39	5		
	More than 40	4		
Education level	Undergraduate	1		
	Master’s	8		
	Doctorate	1		
Years of experience range	3, 20		8.50	5.15
Years of experience group	Less than 5	1		
	Between 5 and 9	6		
	Between 10 and 19	2		
	More than 20	1		
IBCLC certified?	Yes	5		
	No	5		
Role	Midwife nurse	7		
	Physical therapist	2		
	Speech pathologist	1		
Work locations^a^	Hospital	3		
	Clinic	5		
	Residential	10		
	Virtual	9		
Mother?	Yes	7		
	No	3		

^a^Participants were allowed to choose all work locations that applied.

Participants were recruited through social media platforms and snowball sampling (i.e., participants recommended other people working in the field). The inclusion criteria for the study included the following requirements: (1) currently working in the area as a lactation consultant, (2) have more than one year of work experience, (3) have experience performing remote consultations using a PC or smartphone. For the participatory session, we recruited some participants from the contextual interviews (n=5), which followed the same inclusion criteria as the contextual study.

### Data collection and analysis

The contextual study and participatory workshop were conducted using Zoom or WhatsApp, according to the participant’s availability. Due to emergency calls for participants currently at work and time incompatibility (n=3), a few interviews were finished through written text or audio responses using WhatsApp.

The data collected from the contextual interviews consisted of extensive notes (from approximately 6 h of interviews in total), written responses from the participants who did not meet through a call, and 1 hour of audio recording. The participatory workshop was audio-recorded (1 h in total), and a researcher collected extensive notes. All the notes and audio recordings were transcribed in Brazilian Portuguese and then translated into English. After transcribing and proofreading the text by the authors to preserve meaning and context of responses, we performed a thematic analysis on the data using software Nvivo®.

For data analysis, we used thematic analysis techniques (47, 48). The analysis mainly followed an inductive coding approach. During the first stage of analysis, the research team read the transcripts independently and identified repeating ideas or patterns of ideas as themes. The themes are labeled to construct initial codes. We obtained a total of 43 codes (e.g., feeding time and duration, instructions vary with mother’s experience, video database). Descriptive statistics tools were used to summarize the responses, such as in the demographics and contextualization of LCs.

During the second stage, the research team conducted multiple iterations with the codes into themes and subthemes that were categorized and organized into a codebook. The codebook consisted of eight major themes (e.g., important aspects for an effective remote evaluation, drawbacks of remote consultations) and can be found in Appendix A. The research team used the codebook and codewords associated with participants’ comments. Regular meetings were held to discuss the codes assigned to comments for each transcript and to arrive at a consensus. Each code’s frequency is computed and informs how many participants mentioned it within its category.

### Ethical considerations

The Institutional Review Board approved the entire study procedure. Written informed consent was exempt for this study. But all participants provided assent for their participation. Before each session (i.e., interview or focus groups), participant consent verbally about recording the session for transcription purposes. The videos used in the sessions were from one of the author’s database, in which written informed consent was obtained from the individual and the minors’ legal guardians for the publication of any potentially identifiable images or data in this article.

### Positionality

One author is an LC conducting field works with communities in Brazil. Three authors are from Latin America (two are from Brazil) and two are from the United States. Four authors identify as female and one as male. Three authors specialized in technologies for digital health. Two authors work in lactation care and are IBCLC certified with more than 10 years of experience. Three authors are mothers and understand the chestfeeding challenges both as a researcher and as a client of the stakeholder. We all contributed to this paper based on a social justice-oriented (49) and gender-inclusive point of view (50), bringing the term “chestfeeding” instead of “breastfeeding” to provide respect and support to LGBTQI+ parent communities (50).

## Results

Seven participants responded via video call interviews (P1-7), and three responded via text or audio (P8-10). The qualitative interviews focused on getting to know LCs’ field and their interactions with technology for remote consultations until reached saturation of information. In Table 3, we summarize the common findings derived from the participant’s responses during the contextual study. These key findings are then combined into seven major themes and further discussed in detail below through sections “LCs’ relationship with remote care” to “Understanding why LCs are sought for in remote consultations.” From the table, we captured similar responses from participants but also drew out differences that appear to exist between LC’s with different levels of experience and backgrounds.

**Table 3:** Common findings from participants’ results during contextual study.

Participants (*N*)	Mentioned by	Common issues and sentiments expressed
8	P1-4, P7-10	Likes providing remote consultations (RCs)
4	P3-4, P9-10	Started RCs before the COVID-19 pandemic
6	P1-2, P5-8	Started RCs because of the COVID-19 pandemic
4	P2-4, P9	Uses image/video annotation as a guidance for mothers
3	P2-3, P10	Noted benefits to the mother from RCs
1	P2	Uses written step-by-step guide for mothers
5	P2, P4-6, P10	Issues with visualizing the baby in RCs
3	P1-3	Relies on mother mentioning the baby’s sounds in RCs
3	P2-3, P10	Breast injuries, thrush and mastitis are difficult to solve during RCs
5	P2-4, P7-8	Changed communication and instruction techniques for RCs
7	P1-6,P10	LC is mainly about hearing and supporting the mother
5	P3-6, P10	Misses physical contact during consultations
2	P2,P4	Observe diapers is essential for seeing effective chestfeeding
4	P1,P3, P5-6	Baby sounds are important for solving chestfeeding problems

### LCs’ relationship with remote care

All study participants are highly educated in their fields and integrated tools such as computers and smartphones in their LC work. In Table 4, it is sumarized the responses of the questions regarding LC’s current use of technologies, and information about numbers in remote care. The motivations for LCs to start remote consultations were primarily because of the COVID-19 pandemic (n=6) and restrictions to in-person visits. However, some LCs (n=4) mentioned starting before COVID-19 due to some mothers requesting video conferences instead of in-person, especially for patients who live out of the LC’s state. Participant P2 mentioned “having remote consultations before COVID-10, but the number of consultations intensified with the surge of the pandemic.” This statement also agrees with those from P3 and P10. In total, eight LCs said they continue to provide remote lactation services and intend to keep this service modality. However, if the patient requires physical intervention, such as lasertherapy for breast injuries, they might schedule in-person visits according to their needs. Of the two participants who mentioned not being interested in providing remote care, P6, an LC and speech pathologist, informed that “this service [remote consultations] stopped when in-person visits became available respecting the WHO guidelines.” Participant P5, an LC and physical therapist, mentioned trying to provide remote consultations in 2020 but only because the patient was “having chestfeeding problems and was currently with COVID.” However, afterward, she chose not to continue and mentioned not liking this modality.

**Table 4:** LCs’ motivations to provide remote consultations and choice of communication apps.

Category	Sub-category	Participants (*N*)	Mean	SD
When LCs started with remote consultations	Before COVID-19	4		
	During COVID-19	6		
LC’s intent to continue providing remote care	Will continue	8		
	Will not continue	2		
Apps more used for remote consultations^a^	Whatsapp	10		
	Zoom	5		
	Google Meet	2		
	Facetime	1		
Range of remote consultations per week	3, 20		10.25	7.16

^a^Participants mentioned all smartphone and computer applications that applied.

When asked about what platforms they use the most for remote consultations, all participants mentioned using WhatsApp as their main form of communication (n=10) with their clients, but they also shared using other platforms, such as Zoom (n=5), Google Meet (n=2) and Facetime (n=1). More specifically, as described by participant P3 and congruent with the others, “The platform I use the most for communicating and answering questions is WhatsApp.” The participants also mentioned using Zoom and Google Meet for consultations using a computer or laptop, due to the benefits of “being able to view the mother’s positioning and ergonomy,” and also “the mother can use both hands if needed during consultation since they are not holding their phones,” as noted by participants P2, P3, and P7.

The frequency of remote consultations per week varied from 3 to 20 consultations among participants (μ=10.25, σ=7.16). Participants P2, P7, and P8 range between 15 and 20 consultations per week on average, and informed being more focused on remote care for a broader group of parents, correlating to a higher volume of consultations. The participants closer to the lower range of weekly consultations belong to the LCs who are midwife nurses and actively work with birth assistance. Participants P3 and P4 mention that they perform remote consultations with the parents they assist during birth as doulas. These LCs assist the parents starting a few weeks before birth and also chestfeeding guidance after they return home with the baby, being a full-cycle pregnancy assistance approach (from prebirth to postbirth).

### Impressions around remote consultations

The LCs noted various challenges they face with remote consultation even though they recognize the benefits that it may have around access, with some being more positive about the use of remote sessions (P2-4, P7-10), while some would prefer not to (P1, P5-6). P4 notes that because lactation consulting is not a screening procedure, it does not end with a single visit. Instead, it is a process that continues until things are going well and may need to restart when the mother is unsure. In this way, remote consultation allows an LC to have more frequent interface with each mother while supporting multiple mothers at once by removing the need to commute. On the other hand, P1, a midwife with 14 years of experience prior to receiving an IBCLC certification, notes that, in her practice, she relies on long in-person consultations that could last 3 hour and cannot replicate the same experience in a virtual setting.

Benefits such as location and time flexibility (P7-8) for the LC, the possibility of assisting mothers who reside in any location in the country or even in the globe (P3, P7-8, P10), and the possibility of helping more patients (P2, P7, P10) were some benefits mentioned by LCs. Another point discussed in the focus group focused on how LCs had to improve their communication and instruction techniques for an effective consultation, which P2-3 noted as a benefit.

### Virtual settings can be difficult due to dulled senses

**Figure 3:**
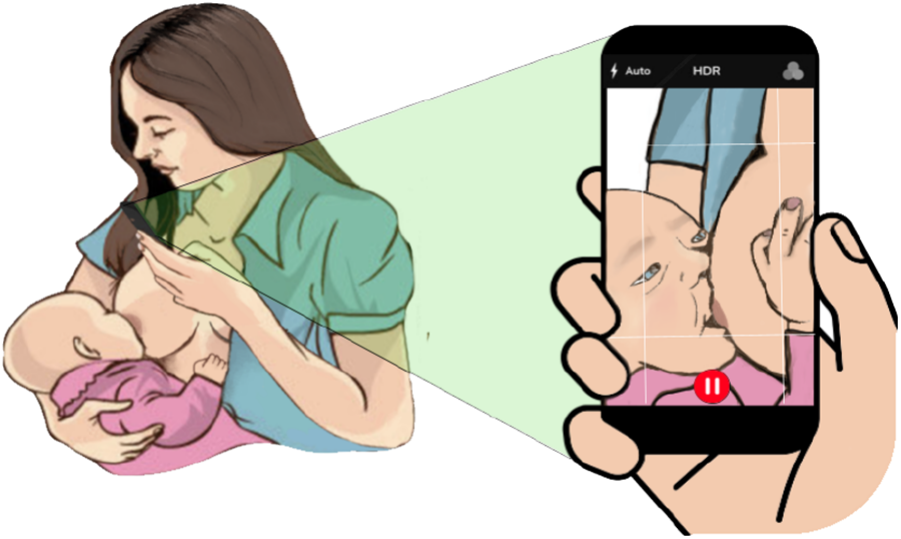
Mother recording herself during chestfeeding.

LCs often ask parents to record a video of their baby chestfeeding by holding the camera from above to show (1) the mother’s holding arm position, (2) baby’s head position with respect to the breast, (3) baby’s mouth shape and movement, and (4) baby’s head and chin tilt (Figure 3). However, in doing so, it is sometimes quite hard to hear the subtle sounds made by the baby during this video call or in a video recording made by the mother. In some situations, LCs mentioned that the mothers would try to describe the situation verbally by describing the sounds the baby is making and what they see that the baby is doing (P1-3). In-person, this would usually be done by visually observing the baby’s mouth movements and hearing sounds that would give clues about milk extraction. At times, LCs would try to circumvent this issue by asking the mother to bring the phone close to the baby’s mouth, but by doing so, they can no longer see the baby’s mouth and position. In addition to the actual observation of the chestfeeding directly, P5 and P6 noted that not being physically present also reduces their ability to look and feel the family’s living contexts (e.g., living conditions, environmental noise, family, and smells).

### Physical manipulation is missing in virtual settings

During a remote session, LCs (P3-6, P10) find it difficult to directly help the mother position the baby’s head or show the mother how to move their hands by direct manipulation. Instead, everything has to be done through verbal description and gesturing. Interestingly, in contrast, P3 mentions that regardless of whether she is in-person or not, she would try to avoid touching the baby and mother to help promote independence of the mother in trying to get the baby to do the right thing. Participants P4-6 and P10 also mentioned missing physical evaluation of the baby’s mouth structure for cases of the lingual frenulum.

This urge for physical demonstration is understandable and noted by other participants, who emphasized that it is difficult to demonstrate gestures to fix latching and positioning. Besides the lack of physical touch, LCs could work around physical demonstration through better communication skills and new procedures for more user-sensible experiences, while still managing to have a large amount of consultations throughout the week, as noted by P3 and is in agreement with the contextual study impressions.

### Remote lactation consulting is useful for parents’ independent learning

Participants P2-3 and P10 noted a benefit of remote sessions that appears to be a by-product of the inability to communicate as easily through verbal, visual, and physical demonstrations. The mothers develop a sense of independence and confidence by trying to understand what the LC is communicating and by doing it themselves. At the same time, the LC had to improve communication and instruction techniques for an effective consultation. The LC cannot easily tell if the mother is doing correctly, so the mother has to describe what they are feeling and seeing more directly. Because the LC is there, the sense of support from a professional is still present (P1-3, P10). P3 elaborated the following about her impressions of the benefits to the mother:

*What I don’t like about remote consultations is that sometimes I feel the need to be hands on to help the mother. But this also is a great learning experience for them, since the person assisted learns to do things for herself, without depending on anyone. (P3)*

In fact, from the inability to communicate as easily through verbal, visual, and physical demonstrations, the mothers develop a sense of independence and confidence by trying to understand what the LC is communicating and by trying to do it themselves.

### Annotations on videos are a way to provide feedback to the mother

**Figure 4:**
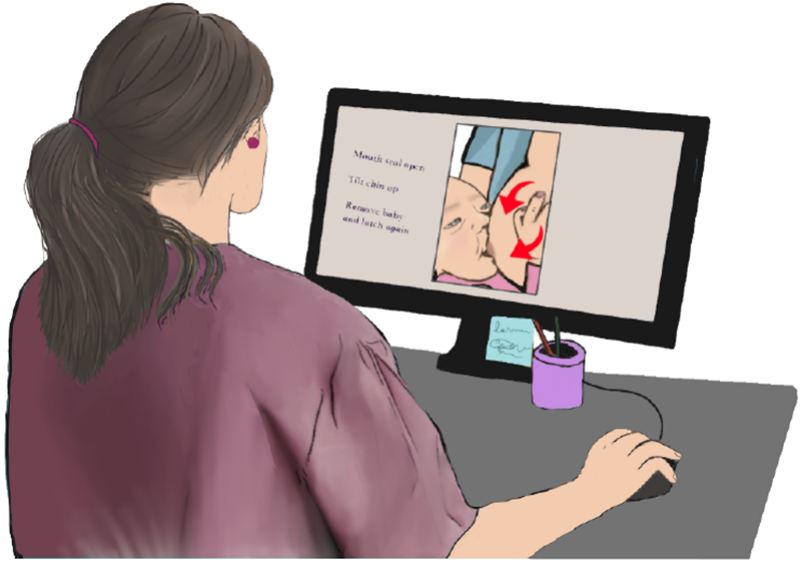
LC correcting a mother’s practice by annotating on chestfeeding videos.

To help mothers get detailed feedback and allow them to review instructions, some LCs would request mothers or family members to take a recording of a chestfeeding session (P2-4, P9). Then annotate with arrows to show what is in the wrong position and how to adjust it on screenshots of specific seconds in the video, or even reinforce that specific movements and noises were correct when answering questions (Figure 4). The LCs that used this approach noted it to be an iterative process sometimes, since the mother would send more videos until the problem is solved.

### Understanding why LCs are sought for in remote consultations

When providing chestfeeding support, there are a variety of cases that LCs can face in their routines, in which some of these cases are rare and require in-person visits or personalized assistance. We asked the participants what the most common chestfeeding questions they get through remote settings are and what are some examples of cases that are solved remotely. We found some cross over in the topics.

One widespread issue in chestfeeding is related to proper latching and positioning of the baby on the breast. Improper latching of the baby can cause pain to the mother, due to soreness or cracked nipples and affect milk production and feeding duration, due to difficulty for mothers to continue the practice if not properly performed from the start. This was the topic most mentioned by LCs (among eight participants) when solving cases remotely, since fixing latching and positioning solves many different problems. Another common topic that LCs received was preparing the mother to transition back into the work routine (brought up by three participants), where they mainly assist mothers with altered milk production and extraction through pumping and storing milk for the baby. These topics are fundamental to the community since they reflect the early interruption of exclusive chestfeeding (11). Finally, LCs also mentioned issues with breast pain as a common topic in their consultations, which unrelated to latching and positioning, can be caused by excessive milk production, breast engorgement, or blocked milk ducts. P2-3 and P10 mentioned this issue in specific as challenging to solve through remote consultations, since it requires evaluating the mother’s breasts or using low-level laser therapy (10) to help heal nipple lesions.

Furthermore, lived experience prior to LC certification training might influence an LC’s perspectives on using technology tools and techniques in their profession. Almost all of our participants were practicing nurses and midwives prior to returning to school to obtain formal international certification. As such, they have years of experience working with mothers already and have internalized and shaped much of their practice. Similarly, those who have been a mother themselves (n=7) explained having a different level of internalization of some of the concepts they learn during lactation studies, and how they approach to parents during consultations.

### Derivations from the contextual study

After conducting the semi-structured interviews, we were able to answer questions about the dynamics of remote consultations for LCs, especially involving the main reasons that mothers reach out to LCs to seek guidance in chestfeeding, and what cases (from the easiest to the most challenging) are solvable through remote consultations.

Since we reached saturation of responses in the contextual study, we idealized a participatory session with some participants present in the semi-structured interviews. The participatory session was based on some of the responses provided during the contextual study, more specifically, focusing on the approach LCs have for solving problems in remote settings and their relationship with technology to provide feedback for parents such as the findings from the subsection ‘Annotations on videos are a way to provide feedback to the mother’. Therefore, for the participatory session, we got a deeper understanding on how the communication channel between LC and parent is started, what are the characteristics in the data exchanged, and what technological features are important to note and use for a proper remote consultation.

During the participatory session, when participants were familiarizing with each other, a discussion started about the participants’ preferences of remote consultations, asked by the moderator. In the session, many of the topics and opinions from participants were congruent with what was found in the contextual study, reinforcing our findings and showing agreement during the focus group. Therefore, the results presented in the previous subsections ‘LCs’ relationship with remote care’, ‘Virtual settings can be difficult due to dulled senses’, and ‘Physical manipulation is missing in virtual settings”, are a combination of the contextual study with the participatory workshop, to avoid repetitions in the results and discussion.

### Making a media platform good enough for chestfeeding evaluation

During the participatory workshop, participants mentioned receiving different types of media from patients in the format of texts, audios, videos, and images with details of the problem the mother has questions about. However, for a good chestfeeding evaluation, the LCs mentioned a few factors that matter the most for them to perform a good evaluation in remote settings. When evaluating the example videos in the focus group, LCs commented on the angle in which the video is filmed, the field of view, the length of the video, and if it is possible to view the initial latching. When the LC can view the baby’s profile (nose, mouth, and chin) and the bodies of both the mother and baby in a video or photo, they can give clues about positioning and how to proceed to change it. In the first video showed in the focus group, P2 mentioned the following:

*We need to see the positioning of the mother and the baby, since the video is recorded too close to the baby we cannot see if we need to position him closer to the mother’s belly, or adjust the arm. We cannot interfere in positioning with no view, and sometimes the positioning changes the latch. (P2)*

Having videos with lengths of at least 1 minute is mentioned as helpful for the participants who provide care based on such media, giving LCs more context. In one of tong, “it’s not enough time to actually understand and identify the context, it’s too short.” P1 added that “shorter videos with no context raise more questions before a proper feedback, therefore it requires more iterations between LC and patient.” The number of iterations depends not only on the context LCs were given, but also how experienced their patients are. If a mother already received instructions and the initial consultation in-person, P9 noted that it is easier and faster to provide feedback and instructions than when the mother has no instructions or they spent little time in the remote consultation. Finally, P3 mentioned the importance of viewing the baby’s initial latch in the breast, which does not only evaluate the moment of feeding but also how a baby placed at the breast impacts the feeding quality and comfort. This will give clues about the shape of the mother’s nipple, how the baby’s lips are positioned, and how deep latched this baby is.

### Preferred elements by professionals when providing chestfeeding feedback

In the technology provocation section, we had the participants reminded of the feedback and suggestions they brought up after watching the videos and how they would use certain features available in mobile platforms to create an artifact with their words and guidance to the original video. ft ted features by the participants were the “adding text instructions or comments” and “audio instructions through voice over.” Participants mentioned adding text in the media as a way to provide step-by-step instructions, and for most cases, they showed interest in using text before any feedback is given, in their communication channels, and asking questions to make sure they know all of the details before proceeding to a formal feedback. Participant P9 mentioned the following regarding using text instructions:

*I believe that text in this example would make me limited in explaining everything, text here would only be used to tell the mother: ‘I will send you an audio’. That would be it. (P9)*

The preference of the participants to use audio instructions prevailed throughout the session. The participants mentioned that using audio on top of the original videos for feedback is time-saving when creating interactive feedback, since it does not require texting or other detailed tasks that needs more interaction with the media for artifact creation. Another positive view from audio recordings is how the participants consider it easier to explain instructions and ideas with more detail in a shorter time; they can also show voice intonation for motivating their patients and get a better personal connection even in remote settings. Here are some other comments received from participants P8, P9, and P3:

*“I prefer audio because it explains better, I feel more comfortable and it optimizes my time” (P8)*,

*“I would record an audio because it’s easier for me to talk about these concepts to the mother” (P9)*,

*“Within the possibility and ease of making an audio over the person’s video I think it would make it a lot easier to the mother understand what I have to say.” (P3)*

After the audio, the participants had several uses and ideas to put into practice when presented with the possibility of using “images with instructions or examples” and “draw or highlight on top of video.” They pointed the importance of using the drawing feature to indicate problems and where specific changes needs to be made (e.g., lip positioning, head rotation, chin placement, and distance). Additionally, the LCs mentioned being familiar with using images with instructions or examples that are useful for cases where more drastic changes are required to fix baby latching and positioning. All the participants confirmed already using example images often in their routines to help their current patients and showed interest in using them to create new artifacts as this paper proposes. As an example of how the participants would use these features, here is what P4 exemplified:

*“I would draw on the image mainly showing the lower lip, because to me it looks like the lower lip is inward in this video, and show example images too.” (P4)*

Another feature option the participants mentioned as useful for them was the idea of creating parallel videos with the original one. This takes the level of interactivity between LCs and patients to the next level since it allows them to display both the current case and an example videos side by side. This specific feature had the participants suggest using it to record videos of themselves demoing with chestfeeding dolls and breast plushies, for a more didactic explanation and personalized to specific cases. Using chestfeeding dolls is a common practice among lactation consultants and is a great way to exemplify conditions remotely. The LCs specified that having the video “with the wrong approach” right next to the “correct approach” gives the possibility to the mother compare herself to the LC’s suggestion and have it easier to fix, as cited by P9 and P2:

*If I could send this mom an example video, she could visually compare herself and see what would be a nutritive suckling and what would not be (P9)*,

*I would start with the video, showing the plushie breast and what the baby is doing on it. We can open this breast to show it from the inside to make her understand the impact of the positioning. (P2)*

Some participants were in favor of using pre-recorded responses in a future tool. They saw benefits in using it, especially when they needed to provide feedback or text instructions related to common problems in chestfeeding. This tool was seen by one participant as interesting and time-saving, especially if the consultant prethinks about the common questions they receive from their patients. Here is what P8 mentioned about the possibility of using such a tool:

*When writing I prefer the pre-written text, therefore I would use the pre-recorded responses if they are available and if the text is related to the condition. (P8)*

Finally, no participants showed interest in using the feature to change the media speed before using the other features to provide feedback. One of the participants brought to the discussion the use of technology tools that, in some cases, are too complex or the number of iterations with the mother has passed a point in which they are required to simply call the mother to explain and help to solve the problem. Additionally, some participants mentioned using other tools to help them optimize their time, such as using speech-to-text features found in current social media applications, and retaining a database of media in which they can share with their patients more easily specific cases in chestfeeding.

### Mixed feelings about using resources for remote care

Throughout the focus group video feedback and technology provocation, we could observe some nuances of opinions related to the use of technologies and specific features to complement the remote experience for lactation consultants and mothers. The participants noted that the use of technology elements could be beneficial for their patient’s understanding and learning, as well as it is a more interactive form of feedback. Most of the participants felt comfortable using technologies and saw it as an item that could benefit their work as LCs, which had them seeing the features as time-saving in case the increased interactivity brings easier understanding on the mother’s side, and help them respond to inquiries quicker with some specific features. On the other hand, some participants who are less familiar with using specific applications and features, found the technology provocation too complex to manipulate and had an idea that, due to this complexity, it might take them extra time to give feedback using the features presented since it will require learning how to use it and practice with real examples. Even though it might be time-consuming and complex, P8 mentioned that still there are benefits of using such tools and how important that is for mothers better and faster understand the instructions LCs provide them. More specifically, when presented with the option of recording videos in parallel to the original video, one solution the entire group found was using pre-recorded instruction videos or materials they found online instead of recording one new video for every single patient they have, especially when it is a common case. Here is what P2 mentioned about how using a diversity of tools and a complete feedback might be impacted by time limitations:

*I would use a chestfeeding doll and record a video, speaking about the ideal world, right?! We can’t always do the ideal feedback with all the tools because sometimes we’re in the car in a rush and we have to record audio only. It’s not always so dynamic. But if on the day I’m working remotely for example, or I am at home, I would record this video talking about the feeding and positioning. (P2)*

## Discussion

Through our formative contextual study and participatory workshop, we identified general themes around remote and virtual lactation consultation. In the following section, we identify a few potential directions that, based on the general themes uncovered in our need-finding, we envision a potential impact on how remote lactation consulting could be conducted.

From the **participatory session**, the authors investigated how LCs analyze and apply their technical skills remotely into possible chestfeeding cases and how they humanize their work by using auditory, visual, and non-verbal cues to provide specialized and caring feedback to their patients. Getting to know the dynamics of Brazilian LCs and how they humanize their consultations and engage with their patients brought exciting insights that are valid to share globally. This work can contribute to positively impacting higher percentages of chestfeeding rates. In addition, once understanding how LCs tuned their eyes and understood the entire story behind an irregular feeding session, we were able to hear from LCs how some tools, among others, would be more beneficial for the mother’s independent learning, feeling of support, and effective guidance, which should be design considerations of any system that deals with vulnerable groups. Drawing from our findings, we discuss the perceived aspects in which technology benefits LCs in general, how we perceive remote consultations as being added in a hybrid modality, and the implications for making it stay in post-pandemic times.

### How technology helps lactation consultants

Our findings show that LCs perceive the benefits of remote lactation consultations during and post-pandemic, not only for themselves as professionals but also for their patients, by helping mother’s independent learning and higher engagement during remote consultations (35, 51). LCs were also able to recognize the challenges of this practice and how each participant could adapt communication skills and organization to accommodate changes and keep patients supported, by using technologies and tools to help manage and organize their remote environment. It is important to note that when developing technology for this community, considerations about keeping lactation–mother partnership strong even when not in-person is essential (24). During the study, LCs noted that technology tools that enable more detailed feedback for parents are more interactive and promote easier understanding on the mother’s side; however, it should be emphasized that these tools should be simple to use by these professionals.

It is necessary to educate the community about the routines of these professionals and understand how tools might be incorporated into LC’s work routine: in Brazil, most of these professionals are not only certified LCs but also nurses, who attend births, have long shifts in-hospital or clinics and perform remote consultations (18, 19). With the busy routine of healthcare providers, bringing easy-to-use, time-saving tools that will deliver effective information for their patients and focus on mitigating prolonged interactions, will not only alleviate the burden on these professionals with the load of patients but also enable a broader understanding of lactation concepts and give more time to these consultants in having new clients, and have broader access by the community. Derived from our findings, we also suggest creating guidelines to keep remote lactation consultations effective. LCs commented on how videos from parents should be recorded at certain distances and angles, and in certain durations to accurately analyze latching and positioning, which will provide LCs with a full view and context for faster feedback.

From our qualitative analysis, the high volume of video conferences with mothers and sharing of audiovisual data for analyzing cases remotely is congruent with related works sharing the preferences of mothers having video remote consultations instead of phone calls (33). Additionally, broader access to tools for midwives and LCs enriches their experience with problem-solving, with more accurate and faster diagnosis, which is essential to keep improving the field to help LCs achieve the recommended chestfeeding percentages by regions (24, 51).

### Potential technology scenarios

Creating technologies to facilitate information access, health support, and lactating care and education is not something new. With the benefits of broader access to mobile devices and even AI to enrich the user’s experience and bring smarter applications, users with access to technical resources have many options when seeking information and guidance, especially in healthcare. However, there is a fine line between using these solutions to bring more inclusion and access to health applications and having people talk to automated devices or chatbots that lack human empathy (52, 53).

Especially in lactating care, the need for LC professionals, even if its remote, should be the primary point of contact due to the richness of experience these professionals have in helping detect problems. LCs provide technical information to patients and guide, nurture, reassure, and help relieve new mothers, who are currently experiencing emotional burden through changes and choices related to chestfeeding (54, 55). Opportunities found in this research are presented in the following items.

• ***Wearable microphone for helping LCs hear better***
  A common issue brought up by all of our participants is this issue around difficulty hearing the sounds produced by the baby. One of the LCs tried dealing with this by bringing the phone’s microphone directly to the baby’s mouth, but that means the phone camera cannot be pointed at the face of the baby. If, however, a separate microphone is worn, say on the nursing bra or even worn on the baby’s skin surface, the sound measurement and visual recording can be decoupled.
• ***Annotation and video review tools for remote sessions***
  Without the physical ability to show and manipulate in-person, LCs noted that virtual sessions could sometimes be frustrating in trying to communicate physical movements and positions that the mother needs to do. The mother would have to hold the phone in one hand to show the baby’s mouth and their other hand’s position while listening to the LC’s descriptions of what to look for, listen for, etc. LCs try to make it easier for the mother by having the mother record a video and then annotate a screenshot with a note as to when in the video this screenshot was taken from. We could consider integrating this interaction into a custom video conferencing interface. A mother could be holding the camera to capture the feeding video snippet, and as this is happening, the LC could try to communicate, as she does now verbally, but, at the same time, could gesture and annotate in-frame. Suppose the mother is confused and needs visual feedback. In that case, they can look at the phone and, in real-time, the interface can replay any part of the session, associated annotations, as well as the LC can make additional annotations to help talk through feedback and clear confusions.
  The ease of control of such an interface would be crucial and potentially needs to be designed such that the LC would actually be controlling the interface since the mother would likely have her hands full already. However, as noted by seasoned LCs in our participant group and by an author of the paper who is a lactation consultant, an important consideration when creating such a user interface is to avoid overreliance on technology interfacing with the mother and the child. When chestfeeding, the mother should spend as little time as possible looking at the phone screen and instead be focusing on the baby. We imagine that the above-suggested interface’s semi-asynchronous feedback after the session could be suited for not being overly intrusive with technology use, but it is important to pay particular attention to actual implementation.
• ***Dashboard for LCs to have better presence of their patients***
  Several LCs mentioned the ability to serve more patients with the reduced time commitment of commuting. Although this was not explicitly mentioned by any LC, the authors noted a potential opportunity that may arise due to this increase in reach of patients. In an ideal trajectory in line with the United Nations Sustainable Development Goals, more parents would be chestfeeding with the help of LCs, a goal that would likely be accelerated by the access to remote lactation consulting. We suspect that this increase in demand could result in individual LCs managing more mother–baby dyads. Back-end dashboard systems that could help LCs manage each mother could be useful. Information managed and organized for each mother such as video recordings with transcribed annotations, relevant metrics such as SSB ratios calculated by an automated AI system, and a direct channel of communication from and to the mother would all be desirable. In particular, we suspect that a key feature of such a system would be for mothers to continuously provide information to the LC, such as any observed pains they are feeling, and record videos out of consultation sessions that a back-end AI system could automatically review. This way, a two-way communication could be established where mothers could reach out through the management tool when they are losing self-confidence for support and LCs could be alerted to a potential decrease in mother self-efficacy to offer just-in-time support.
• ***Building a virtual library of difficult and rare conditions for lactation education***
  One of the potential side effects of lactation consulting becoming heavily remote is the opportunity for many videos to be recorded of chestfeeding sessions. Although we would not imagine, due to the inherently highly private nature of chestfeeding that such videos would not be appropriate for mass distribution, it would be possible that mothers may be willing to approve the use of videos for training of lactation consultants. This is particularly important as described by many participants, difficult and rare conditions can sometimes be difficult to learn in school because there is a lack of educational resources and examples, and it is often learned through practice. However, such practice is difficult in remote settings, and even when learned in-person through hours of practice, the same observational learning may translate better to remote care. By building many examples of different conditions, we could provide better educational content explicitly for the training of remote care delivery. Additionally, with annotations tools such as that described previously, these video samples in the digital library could include relevant notes and descriptions made by trained, practicing lactation experts in context.

### Remote consultations are not a definite solution

From this qualitative study, we found that remote lactation consultations are one of the means but not the main goal. Although some of the participants intend to transition or currently have most of their consultations fully remote, a variety of lactation cases still require in-person visits and physical evaluation from LCs (10, 28). Therefore, we can see remote lactation care being incorporated as two modalities: (1) LC remote consultations being offered as a complement to in-person visits, for follow-up with questions and offering solutions throughout the next days/weeks postpartum, and (2) remote lactation consultations being the only way of contact (especially in rural areas, with patients abroad, as an option for mothers who cannot commute to the clinic) between LCs and mothers, where specific guidance should be provided when there is need for in-person specialty contact.

Drawing from the most common chestfeeding cases that are solved online and being aware of how LCs use procedures or step-by-step guides for their patients, we find opportunities for future research in lactation care. There is space for semi-automated systems, including AI, computer vision, and chatbots for providing online support for specific cases, such as preparing the mother to return to work, verifying baby latching and positioning, and managing low or high milk production and storage.

The main takeaway about remote lactation services is that having technology interventions that would provide a secure environment for mothers to seek guidance, reassurance, and confidence is a necessary option for patients who lack access to such an environment at home (33). Additionally, remote care is a beneficial increment to in-person care, not a replacement, which can be continued beyond early weeks postpartum or even get started before birth with prenatal care.

## Conclusion

This paper detailed a qualitative study that identified how LCs in Brazil perceive remote lactation consultations, what technology elements are most preferred by LCs, and how they would use them when providing remote feedback on chestfeeding for Brazilian mothers. We draw from our results LC’s usage of online tools for providing remote support, their procedures in problem-solving when presented with media such as videos, understanding of the most frequent cases that are solved through remote consultations, how the presention of data is essential for good evaluations, and what technology features are most preferred by LCs when providing chestfeeding instructions with clarity and empathy. We concluded by (1) identifying how LCs have benefited from technologies for remote care and how, in order to keep remote lactation support an option, more integrative and nurturing applications, and solutions should be considered for maternal care, (2) recognizing that the preferences among patients and consultants in remote lactation care will vary based on socio-economic status, type of chestfeeding problem, and familiarity with technology, and (3) understanding how fully remote lactation care may not be the main objective for overall populations, but as a hybrid mode of care that benefits mothers from having both modalities of consultations available to the community. The findings from this paper point out that other regions around the globe might find benefits in applications for chestfeeding support and their needs can differ from the Brazilian community. Future research is required to identify how generalized solutions for remote lactation care can be, especially for different cultures, and possibly evaluate applications for the LC community in the wild.

## Data Availability

The raw data supporting the conclusions of this article will be made available by the authors upon reasonable request.

## Appendix A. Codebook and themes from qualitative analysis

**Table A1:** Codebook created from the qualitative data analysis. There are a total of 8 themes (shown in bold), with its respective codes for each theme.

Theme	Code	Frequency
**Benefits of Remote Consultations (RCs)**		**7**
	Geographic freedom	1
	Helping people from other locations	4
	Learning experience for mother	1
	Time flexibility	1
**Drawbacks of RCs**		**8**
	Difficulty to explain at a distance	2
	Missing hands-on experiences	4
	Issues in being understood	2
**Common questions LCs are asked in RCs**		**12**
	Verifying if latch is correct	2
	Unsure about where is the problem	1
	Baby not taking the breast	1
	Ensuring if baby is chestfeeding properly	1
	Feeding time and duration	1
	Managing milk production	2
	Means to reduce breast pain	4
**Solvable cases through RCs**		**14**
	Preparing the mother to return to work	2
	Milk production and extraction	3
	Latch and positioning	4
	Breast engorgement	2
	Breast pain reduction	2
	Feeding duration	1
**Details of an effective remote evaluation**		**10**
	Visualize initial latch and nipple shape	1
	Instructions vary with mother’s experience	1
	Question mother before providing feedback	1
	Video angle and field of view	4
	Video length	3
**Interactive elements for remote feedback**		**44**
	Adding text instructions or comments	9
	Audio instructions through voiceover	9
	Draw or highlight on top of video	7
	Images with instructions or examples	8
	Pre-recorded responses or instructions	3
	Change video speed for better visualization	0
	Video in parallel with example or tutorial	8
**Feelings about using resources in RCs**		**13**
	Easier for the mother to understand	4
	Editing videos can be time consuming	2
	I am familiar with using tools in RCs	2
	Learn through comparison	1
	Tool habits that are time-saving	2
	Using tools are too complex for me	2
**Other tools used by LCs in remote care**		**16**
	Chestfeeding doll	4
	Call mother to explain	1
	Screeshots	5
	Speech-to-text tool for faster writing	2
	Video database	4
